# Association between retinitis pigmentosa and an increased risk of primary angle closure glaucoma: A population-based cohort study

**DOI:** 10.1371/journal.pone.0274066

**Published:** 2022-09-09

**Authors:** Man-Chen Hung, Yu-Yen Chen

**Affiliations:** 1 Department of Medical Education, Taichung Veterans General Hospital, Taichung, Taiwan; 2 School of Medicine, National Yang Ming Chiao Tung University, Taipei, Taiwan; 3 Department of Ophthalmology, Taichung Veterans General Hospital, Taichung, Taiwan; 4 Wilmer Eye Institute, Johns Hopkins University, Baltimore, Maryland, United States of America; 5 Institute of Public Health and Community Medicine Research Center, National Yang-Ming University, Taipei, Taiwan; 6 School of Medicine, Chung Shan Medical University, Taichung, Taiwan; 7 Department of Post Baccalaureate Medicine, National Chung Hsing University, Taichung, Taiwan; Taipei Medical University, TAIWAN

## Abstract

**Background:**

Retinitis pigmentosa (RP) is the most frequent retinal hereditary dystrophy and result in blindness if progresses. Several case reports have revealed the possible association between RP and primary angle-closure glaucoma (PACG). We conducted a population-based study to explore whether RP significantly increased the risk of PACG development.

**Methods:**

Using the Taiwan National Health Insurance Research Database, we enrolled patients with RP into the RP group from 2001 to 2013 and included a comparison group of 1:4 age- and sex-matched individuals without RP. We performed a Cox regression analysis to estimate the crude and adjusted hazard ratios (HRs) of RP for PACG after adjustment for hypertension, diabetes, hyperlipidaemia, chronic kidney disease, and lens subluxation.

**Results:**

We enrolled 6223 subjects with RP and 24892 subjects for comparison. The mean age of the cohort was 49.0 ± 18.1 years. The RP group had significantly higher percentages of diabetes mellitus, hypertension, and hyperlipidaemia. The cumulative incidence of PACG in patients with RP was 1.61%, which was significantly higher than that in the comparison group (0.81%, p < 0.0001). According to the univariate Cox regression analysis, the hazard of PACG development was significantly greater in the RP group, with an unadjusted HR of 2.09 (95% confidence interval [CI], 1.64–2.65). The increased risk persisted after adjusting for confounders (adjusted HR = 2.18; 95% CI, 1.76–2.72).

**Conclusion:**

This nationwide population-based cohort study showed that people with RP are at a significantly greater risk of developing PACG than individuals without RP.

## Introduction

Retinitis pigmentosa (RP) is a genetic disorder that involves the breakdown and loss of photoreceptor cells. Patients may experience night vision loss at first, followed by peripheral vision loss, optic nerve dysfunction, and blindness. Several studies have reported the possible association between RP and primary angle-closure glaucoma (PACG) [1–4]. The prevalence of PACG among RP patients over 40 years of age was 1.03% in Badeeb’s study in Canada [1], 2.14% in Peng’s study in China [3], and 2.13% in Pradhan’s study in Nepal [4]. The inconsistency might result from the different ethnicities and few cases in these hospital-based studies. Elder et al. even considered that the association between RP and PACG was coincidental because of its rarity [5]. However, some studies have demonstrated that the association between RP and PACG is related to nanophthalmos, cataract, and lens subluxation in RP, which are all predisposing factors of PACG [1,4,6].

Since RP is a rare disease with a worldwide prevalence that varies from 0.014% to 0.04% [7], a population-based study is necessary to enrol a sufficient number of cases. The National Health Insurance Research Database (NHIRD) in Taiwan insures more than 98% of its 23 million residents. Ko et al. used a subset of NHIRD (one million people) and conducted a case–control study over a 15-year period [8]. They found that among the 382 patients with RP, 5 patients (1.3%) had acute angle closure. The case number was still small. Therefore, our study attempted to use the whole population database of NHIRD (23 million people) to include more patients.

The importance of understanding the relationship between RP and PACG is based on two factors. First, an increase in intraocular pressure due to angle-closure may aggravate the visual impairment of RP patients. Second, an appropriate examination should be performed to prevent PACG [9] if RP definitely increases the risk of PACG. Therefore, we conducted a population-based study to investigate whether RP patients have a higher risk of developing PACG.

## Materials and methods

### Data source

This retrospective population-based cohort study was approved by the ethics committee of Taichung Veterans General Hospital (CE21351B). The Taiwan NHIRD is maintained by the Ministry of Health and Welfare and includes all health care records. Diagnoses are registered according to the International Classification of Diseases, Ninth Revision, Clinical Modification (ICD-9-CM) codes. To ensure confidentiality, all the data were deidentified by the Ministry of Health and Welfare and written informed consent was exempt according to the rules of the Institutional Review Board. The database is legal to be used on-site for research purposes after applying for the permission. Application procedures are described online (https://dep.mohw.gov.tw/dos/cp-5283-63826-113.html).

### Inclusion and exclusion criteria

We selected patients with RP (ICD-9-CM codes 362.74) from the NHIRD from January 1, 2001, to December 31, 2013. RP was confirmed by ophthalmologists through a well-acknowledged, standard diagnostic protocol which included retinal bone-spiculae pigments, arteriolar attenuation, and waxy pallor of the optic disk under indirect ophthalmoscopy [10]. Visual field examination revealed a mid-peripheral visual field defect or central island. Electroretinogram showed reduced rod and cone response and delay of time [10,11]. To ensure that the enrolled patients with RP were newly diagnosed in our study, those with RP diagnosed before January 1, 2001 were excluded. In addition, PACG diagnosed before the index date, defined as the date of the first RP diagnosis, were also excluded.

Thereafter, we randomly selected individuals who were never diagnosed with RP and allocated them to the comparison group, which was 1:4 matched with the RP group on age, sex, and index year (year of enrolment).

### Definition of outcome

The two groups were followed until the end of 2013 to determine whether they subsequently developed PACG (ICD-9-CM code 365.2). Patients diagnosed with PACG should show glaucomatous visual field defects or glaucomatous optic neuropathy with primary angle closure [12].

### Identification of comorbidities

We identified comorbidities that were confounders [11] to be adjusted in further statistical analyses. These comorbidities were diabetes mellitus (ICD-9-CM code 250), hypertension (ICD-9-CM code 401–405), hyperlipidaemia (ICD-9-CM code 272), chronic kidney disease (ICD-9-CM code 585), cataract (ICD-9-CM code 366.1), lens subluxation (ICD-9-CM-code 379.32, 379.33, 379.34, 379.39) and nanophthalmos (ICD9-CM code 743.1).

### Statistical analysis

The differences in the demographic/clinical characteristics between the RP and comparison cohorts were analysed by the two-sample t test for continuous variables and the chi-square test for categorical variables. The cumulative incidence of PACG during the follow-up period was also assessed and compared between the two groups.

A Cox proportional hazard model was utilized to estimate the hazard ratio (HR) for the occurrence of PACG according to each variable in the univariate analysis. Two sets of multivariate Cox regression were performed. Covariates adjusted in Model 1 were age, sex, and comorbidities (diabetes mellitus, hypertension, hyperlipidaemia, chronic kidney disease, cataract, and lens subluxation). In Model 2, in order to reduce the possibility of over-adjustment, only diabetes mellitus, hypertension, hyperlipidaemia, chronic kidney disease, and lens subluxation were adjusted as covariates. Comorbidities were considered time-dependent variables to avoid immortal time bias [13]. All analyses were performed by SAS version 9.3 (SAS Institute, Cary, NC, USA). A p value <0.05 was considered statistically significant.

## Results

### Demographic characteristics of the study sample

In the study, a total of 6223 RP patients and 24892 matched comparisons were enrolled. Table 1 displays the demographics and clinical characteristics of the two groups. The mean age in both groups was 49.0 years, with a standard deviation of 18.1 years. The two groups were well matched for age and sex.The RP cohort had a significantly higher prevalence of diabetes (20.4% vs. 17.7%, p<0.0001), hypertension (38.5% vs. 36.3%, p = 0.001), hyperlipidaemia (28.6% vs. 25.7%, p<0.0001), cataract (35.0% vs. 16.2%, p<0.0001) and lens subluxation (2.1% vs. 0.5%, p<0.0001) than the comparison cohort. However, the two cohorts had similar prevalence of chronic kidney disease (5.2% vs. 5.6%, p = 0.26) and nanophthalmos (0.05% vs. 0.02%, p = 0.20). During the study period, the cumulative incidence of PACG in the RP group was significantly higher than that in the comparison group (1.61% vs. 0.81%, p < 0.0001).

**Table 1:** Characteristics of the study subjects.

Variable	RP group n = 6,223	Comparison group n = 24,892	*p-*value
	n (%)	n (%)	
**Age**, year, (mean±SD)	49.0±18.1	49.0±18.1	>0.9999
**Age**, categorical			>0.9999
<40	1,893(30.4)	7,572(30.4)	
40–59	2,494(40.1)	9,976(40.1)	
≥60	1,836(29.5)	7,344(29.5)	
**Sex**			0.63
Male	3,047 (49.0)	12,101 (48.6)	
Female	3,176 (51.0)	12,791 (51.4)	
**Diabetes mellitus**			<0.0001
Yes	1,269 (20.4)	4,396 (17.7)	
No	4,954 (79.6)	20,496 (82.3)	
**Hypertension**			0.001
Yes	2,398 (38.5)	9,036 (36.3)	
No	3,825 (61.5)	15,856 (63.7)	
**Hyperlipidaemia**			<0.0001
Yes	1,781 (28.6)	6,404 (25.7)	
No	4,442 (71.4)	18,488 (74.3)	
**Chronic kidney disease**			0.26
Yes	323(5.2)	1,385(5.6)	
No	5,900(94.8)	23,507(94.4)	
**Cataract**			<0.0001
Yes	2,181(35.0)	4,024(16.2)	
No	4,042(65.0)	20,868(83.8)	
**Lens subluxation**			<0.0001
Yes	132(2.1)	134(0.5)	
No	6,091(97.9)	24,758(99.5)	
**Nanophthalmos**			0.20
Yes	3(0.05)	5(0.02)	
No	6,200(99.95)	24,887(99.98)	
**Outcome = PACG**			
**FU time**, year (mean±SD)	6.1±3.7	6.4±3.7	<0.0001
** PACG during the FU period**	100 (1.61)	202 (0.81)	<0.0001

RP, retinitis pigmentosa; SD, standard deviation; PACG, primary angle-closure glaucoma; FU, follow-up.

### Univariate and multivariate Cox regression

Table 2 shows the univariate and multivariate Cox regression model calculations for HRs of PACG during the 13-year study period. In the univariate analysis, RP, age, female sex, hypertension, diabetes mellitus, hyperlipidaemia, cataract, and lens subluxation all showed a significantly higher risk of developing PACG. The unadjusted HR for PACG was 2.09 times greater in the RP group than in the comparison group [95% confidence interval (CI), 1.64–2.65; *p* < 0.0001].

**Table 2:** Analyses of risk factors for PACG in patients with and without RP.

Predictive variables	Univariate analysis		Multivariate analysis			
	Unadjusted HR (95% CI)	*p-*value	Model 1		Model 2	
			Adjusted HR (95% CI)	*p-*value	Adjusted HR (95% CI)	*p-*value
**RP** (Yes vs. No)	2.09 (1.64–2.65)	<0.0001	2.04 (1.60–2.59)	<0.0001	2.18 (1.76–2.72)	<0.0001
**Age**						
<40	Reference		Reference			
40–60	4.73 (2.87–7.80)	<0.0001	4.55 (2.74–7.57)	<0.0001		
≥60	12.75(7.80–20.71)	<0.0001	12.51 (7.47–20.97)	<0.0001		
**Sex** (Male vs. Female)	0.65 (0.51–0.82)	<0.001	0.71 (0.56–0.90)	<0.01		
**Hypertension**	2.25 (1.79–2.83)	<0.0001	1.08 (0.87–1.35)	0.473	1.31 (0.89–1.46)	0.30
**Diabetes**	2.20 (1.73–2.79)	<0.0001	1.35 (1.03–1.77)	<0.05	1.64 (1.32–2.04)	<0.0001
**Hyperlipidaemia**	1.91 (1.52–2.40)	<0.0001	1.11 (0.86–1.44)	0.428	1.22 (0.93–1.60)	0.15
**Chronic kidney disease**	1.03 (0.66–1.65)	0.855	1.01 (0.80–1.37)	0.738	1.15 (0.86–1.51)	0.36
**Cataract**	7.25 (5.73–9.17)	<0.0001	3.94 (2.99–5.20)	<0.0001		
**Lens subluxation**	2.60 (1.23–5.50)	0.03	1.06 (0.55–2.21)	0.828	1.69 (0.85–3.49)	0.13

Variables included in model 1: RP, age, sex, hypertension, diabetes, hyperlipidaemia, chronic kidney disease, cataract, lens subluxation.

Variables included in model 2: RP, hypertension, diabetes, hyperlipidaemia, chronic kidney disease, lens subluxation.

RP, retinitis pigmentosa; PACG, primary angle-closure glaucoma; HR, hazard ratio; CI, confidence interval.

In Model 1 of the multivariate Cox regression adjusted for all the covariates (age, sex, hypertension, diabetes, hyperlipidaemia, chronic kidney disease, cataract, and lens subluxation), RP remained significantly associated with incident PACG (adjusted HR = 2.04, 95% CI: 1.60–2.59, *p* < 0.0001). Furthermore, both age and female sex were significant risk factors for PACG in the Model 1 multivariate analysis. The adjusted HR for PACG in patients over 60 years of age reached 12.51 (95% CI: 7.47–20.97; *p* < 0.0001) compared to those younger than 40 years of age. Of all comorbidities, diabetes and cataracts were the only two statistically significant risk factors for PACG after adjustment for confounders (adjusted HR = 1.35 and 3.94, respectively).

In order to avoid over-adjustment, we assessed Model 2 which did not include age, sex, and cataract as covariates. In Model 2, RP still had a significant association with incident PACG (adjusted HR = 2.18, 95% CI: 1.76–2.72, *p* < 0.0001). Additionally, diabetes remained a significant risk factor for PACG (adjusted HR = 1.64, 95% CI: 1.32–2.04, *p* < 0.0001).

## Discussion

This 13-year population-based study from the Taiwan NHIRD showed a significantly higher cumulative incidence of PACG in the RP group compared to age- and sex-matched comparisons. After adjustment for hypertension, diabetes, hyperlipidaemia, chronic kidney disease, and lens subluxation in a multivariate Cox regression, RP still significantly increased the risk of incident PACG.

### Prevalence and age of onset for RP

The prevalence of RP varies from 0.014% to 0.04% and depends on ethnicity [7]. In an Asian study conducted by Teo et al., the prevalence of RP was 0.03% in Malay individuals, 0.06% in Indians and 0.09% in Chinese individuals [14]. In the USA and Europe, the prevalence of RP was 0.025% to 0.029% [11], which was similar to that in our study (0.027%; 6 223 in 23 million people). The age of onset for RP might vary with ethnicity and genetics [15]. According to our study, the mean age of the first RP diagnosis was 49.0 years, which was older than that found by Ukponmwan et al. in Nigeria and Tsujikawa et al. in Japan, who reported average ages at RP diagnosis of 36.7 and 35.1 years, respectively [16,17].

### RP and PACG

Several studies reported a relationship between RP and PACG [2,8,18–22]. Anatomically, several ocular characteristics may explain the increased prevalence of angle closure glaucoma in patients with RP. Xu J et al. found that patients with acute primary angle-closure glaucoma (APACG) associated with RP had a significantly greater LT than APACG patients without RP. This is because RP patients have a tendency towards accelerated lens growth [23], and thickened lenses may lead to angle closure glaucoma [24]. Our study revealed a significantly higher prevalence of cataracts in RP patients than in the comparison group. Previous studies also showed that cataracts were more prevalent in RP patients, approximately 43.1% in Western China [25]. In addition, reports found that anterior lens subluxation and ectopia lentis were associated with angle closure glaucoma in RP subjects [26,27]. Our study found that the prevalence of lens subluxation in the RP group was four times greater than that in the comparison group. Lens subluxation also significantly increased the risk of PACG in our univariate Cox regression. However, in multivariate analysis, the significance of lens subluxation disappeared. This may be due to the limited statistical power resulting from the small number of lens subluxation cases in our study.

Nanophthalmos is a predisposing factor for angle closure glaucoma [28]. Ghose et al. documented the simultaneous occurrence of nanophthalmos, angle closure glaucoma and retinitis pigmentosa as the triad of a new syndrome [29]. Mackay, Buys, Wang, Mandel, and Yu et al. also described these associations in case reports [6,18,20,21,30]. Genetic mutations such as membrane frizzled-related protein (MFRP) and rhodopsin (R135 W) have been identified as predisposing factors of this syndrome [6,31]. In our study, the RP group had a higher prevalence of nanophthalmolos than the comparison group, although the number of cases were very low (3 and 5, respectively). Liu et al. proposed that the CRB1 gene may play an important role in the association between RP and PACG [19]. The limitation of our study was the lack of genetic sequencing or laboratory data; therefore, we could not derive the underlying mechanisms of the association between RP and PACG. Further studies combining the Taiwan biobank with the NHIRD are necessary to elucidate whether specific genes or biochemical profiles might explain the relationship between RP and PACG.

### Strengths and limitations

This is the first study to investigate the association between RP and PACG in an entire population on a nationwide scale, with the largest number of cases and a long follow-up period. We used the comprehensive Taiwan NHIRD, in which demographic data, diagnoses, examinations and therapies are recorded and confirmed. In addition, our study not only investigated the association between RP and PACG but also adjusted for the impacts of confounders. Our study revealed a higher prevalence of diabetes, hypertension, and hyperlipidaemia in RP patients, which is compatible with similar non-ocular findings in previous studies [8,11]. We adjusted for the confounding effects of comorbidities. Thus, based on our study, the significant association between RP and PACG has the potential to be a real phenomenon.

Reasons that we ran Model 2 in our multivariate Cox regression are presented here. RP is a set of inherited retinal degenerative diseases that affect photoreceptor and retinal pigment epithelial cells, possibly associated with some ocular complications such as cataract. If cataract is in the correlation pathway between the exposure (RP) and the outcome (PACG), it is not appropriate to be adjusted. Therefore, in Model 2, we did not adjust cataract. Additionally, the study and the comparison cohorts were matched on age and sex. Therefore, we did not adjust age and sex In Model 2 in order to avoid over-adjustment. We still keep Model 1 adjusted for age and sex because in cohort study, even individuals matched on age and sex have different follow-up period; therefore, age and sex might play a role in the Cox regression analysis.

One limitation of our study is, at present, we only have access to NHIRD which is before the year 2013. However, our research still can be regarded as a preliminary study, revealing the association between RP and PACG. Afterwards, we will request the access of recent dataset to see if there are any different statistical results.

Another limitation of our study is the lack of genetic sequencing or laboratory data. Despite of the inherent drawback of database, our large-scale epidemiologic study has revealed the possible relationship between RP and PACG. Based on the findings of our study, laboratory research and further genetic studies will have a role in investigating the underlying mechanisms. Although combining Taiwan biobank with NHIRD is still not allowed now, the proposed legislation for permission is on the way. Otherwise, we can apply international biobank such as UK biobank to elucidate whether specific genes or biochemical profiles may explain the relationship between RP and PACG.

## Conclusions

Our study found that patients with RP have an increased risk of developing PACG. As a result, ophthalmologists should be aware of the risk of PACG when treating patients with RP. Because PACG can result in irreversible optic nerve damage and further deteriorate diminished visual function in RP patients, an appropriate clinical examination is crucial, especially for those who have high risks of PACG (e.g. advanced age, female sex, diabetes, and cataract), to achieve early diagnosis and prompt intervention.
